# Tilia Europæa

**Published:** 1882-04

**Authors:** Ad. Lippe

**Affiliations:** Philadelphia


### CLINICAL BUREAU.

### TILIA EUROPÆA.

Ad. Lippe, M. D., Philadelphia.

The seeds, the inner bark and the flowers of the Linden tree were used by the ancient physicians, and after the common school had forgotten all about it, our inquiring brethren in Vienna constituted a very exhaustive proving with the flowers of this tree. We find careful reports published in the fourth volume of the *Oesterneichische Zeitschrift*, in 1848, embracing all the day-books of all the provers. Very few reports of cures with this well-proved remedy have been published, and we now lay before our colleagues a few symptoms characteristic of this remedy, and sometimes forming a very troublesome accompaniment to a very painful form of disease. When, in rare cases of rheumatic fever, a profuse perspiration breaks out, hope is held out that this perspiration is a beneficial crisis; but, instead of this hope being fulfilled, the sufferer complains of an increasing pain just in proportion as the perspiration increases; motion becomes more painful, so does the swelling of the extremities and joints, and the sore feeling of the whole body increases, there is more thirst and a decided decrease of the urinary secretion. After sleep, especially in the morning, the warm perspiration becomes very profuse. In a recent protracted case of rheumatic fever in an old gentleman, one dose of Tilia CM (Fincke) removed the perspiration and pains at once, and had only to be repeated once in five days. The improvement continued till full health was restored. It will be observed that the Tilia perspiration is "*warm*," differing from the Mercury, which is either cold (forehead) or clammy, oily perspiration, which fails to relieve pain.

